# Sexual Orientation and Risk Factors for Suicidal Ideation and Suicide Attempts: a Multi-centre Cross-Sectional Study in Three Asian Cities

**DOI:** 10.2188/jea.JE20140084

**Published:** 2015-02-05

**Authors:** Qiguo Lian, Xiayun Zuo, Chaohua Lou, Ersheng Gao, Yan Cheng

**Affiliations:** 1 Department of Epidemiology and Social Science, Shanghai Institute of Planned Parenthood Research, Shanghai, China; 2 Department of Epidemiology, School of Public Health, Fudan University, Shanghai, China

**Keywords:** suicide, China, Vietnam, Taiwan, sexual orientation, youth

## Abstract

**Purpose:**

Despite robust empirical and theoretical evidence for higher rates of suicide among lesbian, gay, and bisexual (LGB) youths, little is known about the relationship between suicide and sexual orientation among Asian youths. This study examined differences in prevalence of suicidal ideation and suicide attempts between LGB and heterosexual youths in the cities of Hanoi, Shanghai, and Taipei, China.

**Methods:**

The data are from a community-based multi-centre cross-sectional study conducted from 2006 to 2007, with a sample of 17 016 youths aged 15–24 years from Hanoi, Shanghai, and Taipei. Chi-square test and logistic regression were used to evaluate correlates of suicidal ideation and suicide attempts.

**Results:**

The overall prevalence of suicidal ideation and suicide attempts in the preceding 12 months in LGB youths were both higher than in heterosexual youth (12.8% vs. 8.1% and 4.0% vs. 2.4%, respectively). Stratified by city, the prevalence of suicidal ideation was lowest in Hanoi (2.2%), followed by Shanghai (8.0%) and Taipei (17.0%). Similar trends were observed in the prevalence of suicide attempts, which was lowest in Hanoi (0.3%), followed by Shanghai (1.2%) and Taipei (2.5%). Of note, however, multivariate logistic regression results revealed that LGB youth were at a higher risk for suicidal ideation than heterosexual youth only in Taipei (odds ratio 1.65).

**Conclusions:**

Suicidality is common among Asian youth, with higher prevalence observed in urbanized cities. LGB youths are at greater risk of suicidal ideation than their heterosexual counterparts in Taipei than in the other two examined cities.

## INTRODUCTION

Globally, suicide is one of the leading causes of death among young people.^1^ The economic and human cost of suicidal behaviour to individuals, families, communities, and society makes suicide an enormous public health problem around the world.^2^ Existing evidence shows that suicides are increasingly common among Asian youth.^3^^,^^4^ For example, prevalence of suicide in China is high (23 per 100 000),^5^ and suicide is also the leading cause of death for 15- to 24-year-olds.^6^^,^^7^ However, in contrast to China, Vietnam’s suicide rate (0.98 per 100 000) is dramatically low, while the prevalence rates in Taiwan (6.2 per 100 000) fall between those of Vietnam and China.^8^

Identifying subgroups of youth at elevated risk for suicidal ideation and/or suicide attempts could help direct intervention programmes, because existing studies indicate that suicidal ideation is associated with later attempted suicide, and a history of previous suicide attempts is the greatest predictor of eventual completion of suicide.^2^^,^^9^^,^^10^ In the World Health Organization World Mental Health Survey, carried out in 17 countries, approximately 29% of all ideations end with an attempt, and most notably, the risk for suicide attempt is much higher (OR 117.4–123.1) within the first year of suicide ideation onset compared to those who did not report suicidal ideation.^10^

Studies from around the world have documented that sexual minority (lesbian, gay, and bisexual [LGB]) youths are at dramatically greater risk for suicidal ideation and suicide attempts than their heterosexual peers.^11^^–^^13^ A report from the United States suggested that LGB youths are 2 to 3 times more likely to report suicidal ideation and suicide attempts.^14^ Another paper, using data from a school-based survey of adolescents, reported that youth with same-sex attractions were more than twice as likely as those with only opposite-sex attractions to attempt suicide.^15^ In a sample of 9th- to 12th-grade students in the United States, youths who identified as LGB were 3 times more likely than their heterosexual peers to have attempted suicide in the past year.^16^

The epidemiology of suicide is multi-factorial and complex. Beside school environments, social support plays an importance role in the suicide prevention of youth.^17^^,^^18^ A strong support system of family and friends may act like a protective mental shield against suicidal thoughts. A number of studies have documented that strong family connectedness provided significant protection against suicidal ideation and suicide attempts for LGB participants.^17^^–^^20^

However, despite a growing body of research conducted in Western countries,^17^^–^^20^ relatively little attention has been given to the problem of suicidal behavior in sexual minority youths living in Asian cities, and the epidemiology of suicide and its related factors in Eastern countries differs from that in Western countries because of stronger intergenerational ties and the unique Confucian culture.^4^^,^^21^^,^^22^ The present study examines the relationship between sexual orientation and suicide in 3 Asian cities using a large community-based sample. The cultures of Taipei, Shanghai, and Hanoi are all rooted in Confucian values, though these three cities are experiencing different stages of economic and social transitions, with Taipei being the most industrialized city, followed by Shanghai and Hanoi.

Understanding the relationship between sexual orientation and suicide risk might contribute to recognition of vulnerable youth populations and lead to effective and targeted preventive interventions. On the basis of prior research, the goals of this study are as follows: (1) determine the 12-month prevalence of suicidal ideation and suicide attempts among youth 15–24 years of age with different sexual orientations in Hanoi, Shanghai, and Taipei; and (2) assess the hypothesis that suicidal ideation and suicide attempts are associated with sexual orientation and other predictors (drinking, smoking, family history of suicide, living situations, and parental relationships).

## METHODS

### Study details

The present study was conducted in Taipei, Shanghai, and Hanoi, China, by collaborators from the Johns Hopkins Bloomberg School of Public Health, the Population and Health Research Centre in Taiwan’s Bureau of Health Promotion, the Shanghai Institute of Planned Parenthood Research, and the Hanoi Institute for Family and Gender Studies. Data for this study come from a 2006 community-based cross-sectional survey of 17 016 youths 15 to 24 years of age which was conducted in urban and rural areas of Taipei, Shanghai, and Hanoi, with 4354, 6299, and 6363 respondents, respectively.

### Sampling method

The sampling methodology used in this study has been described in detail previously.^23^ A multistage sampling method was employed to ensure a representative sample within each site. In Hanoi and Shanghai, the study participants were sampled from both private residences and group living facilities. In Taipei, students were recruited from schools, with a small non-student subsample interviewed where they lived. The questionnaire was developed by the research team, translated, back-translated, and pilot tested at each site before the survey. Interviewers in 3 sites all received extensive training. Most of the interview was conducted face-to-face, and for sensitive questions, computer-assisted self-interview was adopted after informed consent was obtained. The interviews were anonymous, and data were confidential and accessible only to the researchers. Ethics approval was obtained from the Committee on Human Research at the Johns Hopkins University as well as the collaborating local cites.

### Measures

The questionnaire was constructed according to previously validated scales obtained from the review of the literature after being discussed by researchers from Johns Hopkins University and partners from three local organizations.

*Sexual Orientation.* Sexual orientation was assessed through the question (scored on a 5-point Likert scale): “Which of the following best describes your feelings? (Here we are talking about attraction, not having sex.)”, with possible responses of (1) 100% heterosexual (attracted only to people of the opposite sex); (2) Mostly heterosexual (mostly attracted to people of the opposite sex); (3) bisexual (equally attracted to men and women); (4) Mostly homosexual (mostly attracted to people of my same sex); or (5) 100% homosexual (attracted only to people of my same sex).^24^^,^^25^ Items 1 and 2 were collapsed into a category of non-LGB youth, and items 3 through 5 were collapsed into a category of LGB youth.^14^

*Suicidality.* The questionnaire included two questions concerning suicide.^3^ One question assessed suicidal ideation: “During the past 12 months, have you ever thought about hurting yourself physically or killing yourself?”. Another question assessed suicide attempt: “During the past 12 months, did you ever attempt suicide?”.

*Demographic characteristics.* The questionnaire included a wide variety of demographic characteristics, including age, gender, sexual orientation (LGB or Non-LGB), living situations (live with parents or others [relatives/friends/alone]), cigarette use in past month (yes or no), alcohol use in past month (yes or no), and other demographic characteristics, including education level, wealth, marital status, and household economic status.

*Family characteristics.* Scales were constructed to measure family characteristics. The ‘mother and father relationship’ construct consisted of seven items, each measuring aspects of parent-child relationships, including parental support and monitoring. Participants were asked how often (never, sometimes, often, or always) their primary female or male caregiver had exhibited certain parental behaviors when they were 13–14 years old (ie, showed you that he or she loved you, was interested in how you were doing, expected you to do your best). A composite score was obtained by summing the items, with higher scores reflecting higher parental support and monitoring. Cronbach’s alpha values were 0.69 and 0.73 for the mother and father relationship scales, respectively.^3^ Family history of suicide was measured by the question: “Has anyone else in your family ever tried to kill themselves?” (yes or no).

### Statistical analysis

Contingency tables were used to describe the prevalence of demographics and primary outcomes among participants from the three cities. In the interest of statistical power and easy interpretability, all variables with multiple response options were dichotomized for multivariate analysis. Odds ratios (ORs) and 95% confidential intervals (CIs) for the association between suicidality (suicidal ideation and suicide attempts) and sexual orientation were estimated using logistic regression models with adjustment for potential confounding by age, gender, family history of suicide, family structure, parent-child relationship, smoking status, and drinking status. Heterosexuals (non-LGB) served as the reference group. Statistical significance was set at *P* < 0.05. All data analyses were performed with Stata/SE 12 (StataCorp, College Station, TX, USA), using Stata “SVY” commands, which fit statistical models for the clustered sampling design of the survey.^26^

## RESULTS

### Demographic characteristics of respondents

The weighted descriptive results for demographic characteristics are summarized in Tables 1 and 2. The sample for this study includes 17 016 youth aged 15–24 years, with 6393 Vietnamese, 6299 Chinese, and 4354 Taiwanese participants. The proportion of Shanghai respondents aged 15–19 years (56.4%) was higher than that of Taipei (49.0%) and Hanoi (46.6%). Significant differences were also observed among the three cities in terms of LGB sexual orientation (8.4% overall): fewer Hanoi respondents (3.9%) identified themselves as LGB than their peers in Taipei (8.6%) and Shanghai (10.4%). The majority of respondents (69.8%) reported living with their parents.

**Table 1.** Characteristics of the study population

Characteristics	Cities			Total (*n* = 17 016)

	Hanoi (*n* = 6363)	Shanghai (*n* = 6299)	Taipei (*n* = 4354)	
Age (years), % (*n*)				
15–19	46.59 (3177)	56.42 (3517)	48.98 (2274)^a^	50.78 (8968)
20–24	53.41 (3186)	43.58 (2782)	51.02 (2080)	49.22 (8048)
Gender, % (*n*)				
Male	51.63 (3135)	49.25 (3060)	50.96 (2176)	50.59 (8371)
Female	48.37 (3228)	50.75 (3239)	49.04 (2178)	49.41 (8645)
Sexual orientation, % (*n*)				
Non-LGB	96.11 (6155)	89.59 (5705)	91.44 (3997)^b^	92.47 (15 857)
LGB	3.89 (208)	10.41 (595)	8.56 (357)	7.53 (1159)
Living conditions, % (*n*)				
Parents	72.72 (4699)	66.18 (3641)	70.70 (3080)	69.81 (11 420)
Others	27.28 (1664)	33.82 (2658)	29.30 (1274)	30.19 (5596)
Suicidal ideation, % (*n*)				
No	97.79 (6207)	92.00 (5795)	83.04 (3608)^b^	91.59 (15 607)
Yes	2.21 (159)	8.00 (504)	16.96 (746)	8.41 (1409)
Suicide attempts, % (*n*)				
No	99.68 (6339)	98.76 (6209)	93.08 (4069)^b^	97.50 (16 617)
Yes	0.32 (24)	1.24 (90)	6.92 (285)	2.50 (399)

LGB, lesbian, gay, and bisexual.

Wald (Pearson) test, ^a^*P* < 0.05, ^b^*P* < 0.001.

**Table 2.** Characteristics of the study population, stratified by sexual orientation

Characteristics	Hanoi (*n* = 6363)		Shanghai (*n* = 6299)		Taipei (*n* = 4354)		Total (*n* = 17 016)	
			
	Non-LGB	LGB	Non-LGB	LGB	Non-LGB	LGB	Non-LGB	LGB
Age (years), % (*n*)								
15–19	50.50 (3108)	33.17 (69)	54.81 (3127)	65.66 (390)	51.76 (2069)	57.42 (205)	50.50 (8304)	54.25 (664)^a^
20–24	49.50 (3047)	66.83 (139)	45.19 (2578)	34.34 (204)	48.24 (1928)	42.58 (152)	49.50 (7553)	45.75 (495)
Gender, % (*n*)								
Male	49.99 (3077)	27.88 (58)	48.96 (2793)	44.95 (267)	50.76 (2029)	41.18 (147)	51.32 (7899)	41.58 (472)^b^
Female	50.01 (3078)	72.12 (150)	51.04 (2912)	55.05 (327)	49.24 (1968)	58.82 (210)	48.68 (7958)	58.42 (687)
Living conditions, % (*n*)								
Parents	74.70 (4598)	48.56 (101)	57.46 (3278)	61.11 (363)	70.58 (2821)	72.55 (259)	70.09 (10 697)	66.39 (723)^b^
Others	25.30 (1557)	51.44 (107)	42.54 (2427)	38.89 (231)	29.42 (1176)	27.45 (98)	29.91 (5160)	33.61 (436)
Suicidal ideation, % (*n*)								
No	97.51 (6002)	97.12 (202)	92.15 (5257)	90.57 (538)	83.81 (3350)	72.27 (258)	91.95 (14 609)	87.21 (998)^b^
Yes	2.49 (153)	2.88 (6)	7.85 (448)	9.43 (56)	16.19 (647)	27.73 (99)	8.05 (1248)	12.79 (161)
Suicide attempts, % (*n*)								
No	99.64 (6133)	99.04 (206)	98.69 (5630)	97.47 (579)	93.72 (3746)	90.48 (323)	97.63 (15 509)	95.98 (1108)^b^
Yes	0.36 (22)	0.96 (2)	1.31 (75)	2.53 (15)	6.28 (251)	9.52 (34)	2.37 (348)	4.02 (51)

LGB, lesbian, gay, and bisexual.

Wald (Pearson) test, ^a^*P* < 0.01, ^b^*P* < 0.001.

### Prevalence of suicidal ideation and suicide attempts across cities and sexual orientations

Findings for prevalence are presented in Tables 1 and 2. The prevalence of suicidal ideation in the preceding 12 months among youth aged 15–24 years varied significantly across the 3 cities (2.2% in Hanoi, 8.0% in Shanghai, and 17.0% in Taipei), with an overall prevalence of 8.4%. The prevalence of suicide attempts in the preceding 12 months was lower than that of suicidal ideation (2.5% overall) but shares the similar trend of being lowest in Hanoi (0.3%), followed by Shanghai (1.2%) and Taipei (6.9%). The prevalence of suicidal ideation and suicide attempts among LGB respondents were both significantly higher than among their heterosexual counterparts (12.8% vs. 8.1% and 4.0% vs. 2.4%, respectively).

### Multivariate results: socio-demographic correlates of suicide

As illustrated in Table 3, there were many risk and preventive factors in common between suicidal ideation and suicide attempts, including younger age, female gender, family history of suicide, cigarette smoking, and drinking. Of special note, positive parental relationships were preventive factors of suicidal ideation and suicide attempts.

**Table 3.** Multivariate logistic regression of correlates of suicidal ideation and suicide attempts

Characteristics	Suicidal ideation, OR (95% CI)				Suicide attempts OR (95% CI)			
	
	Hanoi (*n* = 6363)	Shanghai (*n* = 6299)	Taipei (*n* = 4354)	Total (*n* = 17 016)	Hanoi (*n* = 6363)	Shanghai (*n* = 6299)	Taipei (*n* = 4354)	Total (*n* = 17 016)
Sexual orientation (Ref = Non-LGB)	0.79 (0.32–2.00)	0.88 (0.57–1.35)	1.65 (1.12–2.43)^a^	1.40 (1.03–1.89)^a^	1.50 (0.37–6.14)	1.36 (0.68–2.71)	1.17 (0.78–1.75)	1.34 (0.96–1.88)

Age group (Ref = 15–19 years)	0.57 (0.43–0.75)^a^	0.74 (0.57–0.96)^a^	0.46 (0.38–0.56)^c^	0.55 (0.47–0.66)^c^	0.81 (0.33–1.99)	1.10 (0.61–1.98)	0.63 (0.45–0.88)^b^	0.70 (0.50–0.98)^a^
Gender (Ref = Male)	2.36 (1.31–4.27)^b^	1.53 (1.15–2.03)^b^	1.85 (1.49–2.30)^c^	1.82 (1.45–2.28)^c^	2.54 (0.67–9.57)	1.54 (0.77–3.10)	1.97 (1.45–2.69)^c^	2.12 (1.43–3.15)^c^
Family history of suicide (Ref = No)	4.34 (1.68–11.21)^b^	3.63 (1.95–6.78)^c^	2.06 (1.60–2.65)^c^	3.81 (3.01–4.83)^c^	3.84 (0.88–16.84)	4.33 (1.78–10.54)^b^	2.08 (1.35–3.20)^b^	4.62 (3.10–6.88)^c^
Living conditions (Ref = Parents)	0.89 (0.52–1.51)	0.82 (0.61–1.09)	0.79 (0.66–0.96)^a^	0.83 (0.67–1.03)	1.02 (0.48–2.13)	1.00 (0.59–1.72)	0.90 (0.67–1.22)	0.86 (0.64–1.16)
Mother relationship	0.92 (0.83–1.02)	0.99 (0.94–1.05)	0.97 (0.93–1.00)	0.95 (0.92–0.98)^c^	0.78 (0.56–1.07)	1.00 (0.86–1.17)	0.93 (0.88–0.98)^b^	0.91 (0.86–0.96)^b^
Father relationship	0.86 (0.77–0.95)^b^	0.91 (0.86–0.96)^b^	0.97 (0.94–1.00)	0.92 (0.90–0.95)^c^	0.95 (0.70–1.30)	0.88 (0.76–1.01)	0.96 (0.92–1.00)	0.92 (0.87–0.97)^b^
Currently smoking (Ref = No)	1.49 (0.61–3.65)	1.44 (1.02–2.02)^a^	1.69 (1.24–2.31)^b^	1.63 (1.26–2.13)^c^	0.71 (0.11–4.61)	1.74 (0.73–4.17)	2.15 (1.48–3.12)^c^	2.14 (1.43–3.20)^c^
Currently drinking (Ref = No)	1.97 (1.38–2.82)^c^	1.33 (0.98–1.82)	1.46 (1.20–1.76)^c^	1.46 (1.23–1.74)^c^	1.58 (0.38–6.47)	2.09 (1.22–3.58)^b^	1.54 (1.09–2.15)^b^	1.73 (1.30–2.32)^c^

CI, confidence interval; LGB, lesbian, gay, and bisexual; OR, odds ratio; Ref, reference group.

^a^*P* < 0.05, ^b^*P* < 0.01, ^c^*P* < 0.001.

LGB youths were more than 1.4 times as likely to report suicidal ideation (OR 1.40; 95% CI, 1.03–1.89) compared with their heterosexual counterparts. Family history of suicide was a predictor of suicidal ideation (OR 3.81; 95% CI, 3.01–4.83) and suicide attempts (OR 4.62; 95% CI, 3.10–6.88). Not living with parents had no influence on the odds of suicidal ideation or suicide attempts, while having a good relationship with parents was associated with a 5% to 9% reduction in the odds of suicidal ideation (OR 0.92; 95% CI, 0.90–0.95) and suicide attempts (OR 0.91; 95% CI, 0.86–0.96). Finally, more females experienced suicidal ideation (OR 1.82; 95% CI, 1.45–2.28) and suicide attempts (OR 2.12; 95% CI, 1.43–3.15), and older participants reported suicidal ideation (OR 0.55; 95% CI, 0.47–0.66) and suicide attempts (OR 0.70; 95% CI, 0.50–0.98) less often than younger ones.

When stratifying by city, there were more commonalities in suicidal ideation and more differentials in suicide attempts. Study participants who were younger, female, had a family history of suicide, smoked cigarettes, and drank alcohol were more likely to report suicidal ideation. LGB youths in Taipei were more likely to report suicidal ideation compared with non-LGB youths (OR 1.65; 95% CI, 1.12–2.43) while no association was found among the LGB youths in the other two cities. Further, only youths in Taipei shared the same pattern in suicide attempts (Table 3).

## DISCUSSION

To our knowledge, this is the first study with a large and community-based sample in a non-Western context to explore the relationship between sexual orientation and suicide. We assessed this relationship in three Asian cities with shared Confucian values but in different stages of economic and social transitions.

We found that the prevalence of suicidal ideation and suicide attempts was highest in Taipei, followed by Shanghai and Hanoi, which may reflect the fact that Taipei and Shanghai are much more urbanized communities than Hanoi, where youths may feel isolated, anonymous, and live in much higher population densities. Urbanization may influence suicidal ideation and suicide attempts through depression, since existing studies suggest that a high level of urbanization is associated with increased risk of suicide for both women and men, although many positive aspects of urbanization help to reduce suicide rates, including improved job opportunities and easier access to social and psychiatric services.^27^^,^^28^ Another possible reason for the high percentage of reported suicidal ideation and suicide attempts in Taipei is that most of the respondents sampled in Taipei were students, while the other two cities included both in-school and out-of-school youth. In school-based studies that found high prevalence of suicidal suicidal ideation and suicide attempts, many school-related factors, including academic stress, dissatisfaction with school, failures in exam, boarding conditions, and low school connectedness, were leading causes of suicidal ideation and suicide attempts.^29^^–^^31^

The low prevalence of individuals that identify as LGB in Hanoi (4%) may be due to the fact that Hanoi is the political capital of Vietnam and is more conservative than Ho Chi Minh City, the economic center of Vietnam, which is more urbanized; the prevalence of LGB self-identification in Ho Chi Minh City might be closer to the prevalence in Shanghai (10%) and Taipei (9%).

In this multi-centre community-based cross-sectional study, we observed that, consistent with prior findings,^11^^–^^15^ there was regional variation in the prevalence of suicidal ideation and suicide attempts, with higher prevalence observed in more urbanized cities (Table 1), and the prevalence of LGB individuals was higher (at varying levels) across the three cities in a descriptive analysis (Table 2). However, the adjusted risk of sexual orientation for suicidal ideation was significant only in Taipei (Table 3), which may mean that LGB youth cope with negative feelings in different ways from their peers.^18^^,^^32^ Besides potential differences in coping strategies, there are two other possible explanations for the lack of a significant relationship between sexual orientation and suicidal ideation in Shanghai and Hanoi. The size of the subgroups identifying as LGB and reporting suicidal ideation and/or suicide attempts may have been insufficient, especially in Shanghai and Hanoi, leading to wider confidence intervals. The other explanation is that Confucian values may act as a protector against suicide, with their influence weakening with the progression of urbanization. These three cities with deep-rooted Confucian values have been open to outside influences for different periods and in different ways and, in consequence, their traditional cultures may have changed to different extents.^3^ Taipei is the most industrialized city, hence the protective role against suicide is weakest.

The findings of the present study show that family history of suicide is one of the key risk factors for youth suicide, which is consistent with prior literature.^3^^,^^33^^,^^34^ In our multivariate logistic regression, family history of suicide was the strongest predictor of both suicidal ideation and suicide attempts across the three cities. Data from the study revealed that better parental relationships were weak protective factors of suicide, although previous studies documented that family environment played an important role in reducing the risk of suicide, especially among LGB youths.^17^^,^^20^ Further, we observed that in Taipei, not living with one’s parents was protective against suicidal ideation. Living away from high parental expectations and/or the freedom to live without parental restrictions may be a possible explanation for this counter-intuitive finding,^3^ which has important implications for both public health and education policies. Another possible explanation for this counter-intuitive finding is that the parental support and monitoring scale might be interpreted differently between Asian Confucian cultures and Western cultures. To some extent, this reflects public attitudes in Asia: ingrained Confucian values, which stress the importance of a son’s continuation of the family line, may influence rates of suicidal ideations and suicide attempts among LGB youths.^35^

Similar to another study,^36^ our findings suggested that cigarette use and current drinking might be associated with suicide, although the relationship may be bidirectional. Youths who smoked in the preceding month showed a higher prevalence of suicidal ideation and suicide attempts than those who did not, although a study conducted in 2003 did not report tobacco consumption itself as a risk factor for suicide.^37^

Some limitations should be borne in mind when interpreting these results. First, the study design was cross-sectional, which does not permit causal inference. Second, although the questions that measure sexual orientation and suicide have been widely used in previous studies,^14^^,^^16^^,^^32^^,^^38^ their validity and reliability in Asian contexts remain unclear, partly due to social stigmas and other pressures in Confucian culture. Third, embarrassment, fear of discovery, and anxieties associated with sexual questions in general may also influence answering such questions,^38^ which could introduce information bias. However, computer-assisted self-interviewing software was adopted in this study, which created a sense of anonymity and may help limit response bias when gathering sensitive information dealing with behaviours perceived to be socially undesirable.^39^ Fourth, we did not assess characteristics of those who actually died by suicide, which may have introduced survivor bias. Finally, the study was conducted in three cities in Asia, which implies that the external validity of our findings merits further consideration.

### Conclusion

This multi-centre community-based analysis demonstrated a higher prevalence in suicidality in urbanized cities but an elevated adjusted risk among LGB youth only in Taipei. Our results also highlight the importance of targeting tobacco or alcohol users, as well as youths with family risk factors, for intervention programs.
